# Plant and Fungal Genome Editing to Enhance Plant Disease Resistance Using the CRISPR/Cas9 System

**DOI:** 10.3389/fpls.2021.700925

**Published:** 2021-08-10

**Authors:** Narayan Chandra Paul, Sung-Won Park, Haifeng Liu, Sungyu Choi, Jihyeon Ma, Joshua S. MacCready, Martin I. Chilvers, Hyunkyu Sang

**Affiliations:** ^1^Department of Integrative Food, Bioscience and Biotechnology, Chonnam National University, Gwangju, South Korea; ^2^Kumho Life Science Laboratory, Chonnam National University, Gwangju, South Korea; ^3^Department of Plant, Soil and Microbial Sciences, Michigan State University, East Lansing, MI, United States

**Keywords:** CRISPR/Cas9, plant resistance, fungal and oomycete pathogens, plant genome editing, fungal and oomycete genome editing

## Abstract

Crop production has been substantially reduced by devastating fungal and oomycete pathogens, and these pathogens continue to threaten global food security. Although chemical and cultural controls have been used for crop protection, these involve continuous costs and time and fungicide resistance among plant pathogens has been increasingly reported. The most efficient way to protect crops from plant pathogens is cultivation of disease-resistant cultivars. However, traditional breeding approaches are laborious and time intensive. Recently, the CRISPR/Cas9 system has been utilized to enhance disease resistance among different crops such as rice, cacao, wheat, tomato, and grape. This system allows for precise genome editing of various organisms via RNA-guided DNA endonuclease activity. Beyond genome editing in crops, editing the genomes of fungal and oomycete pathogens can also provide new strategies for plant disease management. This review focuses on the recent studies of plant disease resistance against fungal and oomycete pathogens using the CRISPR/Cas9 system. For long-term plant disease management, the targeting of multiple plant disease resistance mechanisms with CRISPR/Cas9 and insights gained by probing fungal and oomycete genomes with this system will be powerful approaches.

## Introduction

An increasing human population needs to have sufficient food supplies. With a projected global population in 2050 of 9.2 billion, this creates a significant increase in demand for food. Not only is global food security challenged by an increasing population and climate change, but by a multitude of evolving, emerging, and introduced plant pathogens (Anderson et al., 2004; Bebber and Gurr, 2015). The most destructive plant pathogens are the fungi and oomycetes, which are taxonomically distinct but have similar filamentous growth and host infection structures (Dong et al., 2015). These pathogens can destroy crops in a short period of time and cause severe famine such as the Irish potato famine caused by the oomycete pathogen *Phytophthora infestans* (Turner, 2005; Fones et al., 2020) and the Bengal famine caused by the rice brown spot fungal pathogen, *Cochliobolus miyabeanus* (Chakrabarti, 2001; Corredor-Moreno and Saunders, 2020). The use of pesticides, cultural, and cultivation practices can provide a level of protection from plant disease. However, pesticides can cause severe environmental devastation and are rapidly losing efficacy due to pathogen evolution, and they are often strictly regulated to minimize unwanted side effects (McDonald and Stukenbrock, 2016; Vannier et al., 2019; Esse et al., 2020; Tyagi et al., 2021). The development of plant varieties with inherent disease resistance through breeding provides an environmentally friendly and often complimentary approach to plant disease management (Sánchez-Martín and Keller, 2019). Plant breeding for disease-resistance generally relies on identifying plants that carry considerable disease resistance traits, the growth, and development of breeding candidates in a disease-conducive setting, and finally, selecting disease resistance individuals that retain yield.

Current plant breeding methods to obtain productive, disease free, nutritious, and safe crops utilize both conventional and molecular methods (Yin and Qiu, 2019; Kaiser et al., 2020; Tyagi et al., 2021). These approaches have been developed, modified, and re-examined over time. Conventional breeding strategies include pure line selection, pedigree, interspecific hybrids, and back-cross methods. Alternatively, molecular approaches contain variation within multiple genes, marker-assisted breeding, transgenic and tissue culture methods, gene silencing, and plant susceptibility alleles (Capriotti et al., 2020). Conventional breeding, mutation breeding, and transgenic technology have been successful over the last few decades, but several limitations exist, including: (i) intensive labor cost, (ii) inherent genetic variation within plant populations, and (iii) transfer of undesirable genes or traits along with desired resistance genes (Gao, 2018; Ahmad et al., 2020).

In recent years, new breeding technologies such as meganucleases (MNs), zinc-finger nucleases (ZFNs), transcription-activator-like (TAL) effector nucleases (TALENs), and clustered regularly interspaced palindromic repeats (CRISPR) and CRISPR-associated protein 9 (Cas9) endonucleases have been developed to overcome these limitations (Townsend et al., 2009; Gao et al., 2010; Cermak et al., 2011; Li et al., 2012, 2013; Nekrasov et al., 2013; Shan et al., 2013). These new breeding technologies allow precise genetic modifications of single or multiple gene targets in plants (Borrelli et al., 2018). MNs, ZFNs, TALENs and CRISPR/Cas9 are sequence-specific nucleases that cleave target DNA. Double-stranded breaks present in DNA are repaired by host cell repair mechanisms such as homology-directed repair (HDR) or non-homologous end-joining (NHEJ), but small indels (insertions/deletions) can occur within the target region (Langner et al., 2018). Although MNs, ZFNs, and TALENs were applied before the introduction of CRISPR/Cas9, the three techniques have not been widely utilized for plant breeding due to the need for complex protein engineering systems. The increasing number of recent reports for plant genome editing using the CRISPR/Cas9 system indicates that this approach is practical to apply due to its higher success rate and ease of use. The CRISPR/Cas9 system has been applied to enhance multiple beneficial traits of plants including the improvement of disease resistance (Borrelli et al., 2018; Langner et al., 2018; Yin and Qiu, 2019; Zaynab et al., 2020). In addition to applications in plants, genes encoding proteins that interact between host plants and fungal and oomycete pathogens have been targeted by CRISPR/Cas9 to elucidate the underlying molecular mechanism of host-pathogen recognition and to generate screening systems for disease resistance (Fang and Tyler, 2016; Li et al., 2018).

To date, most reviews of plant disease resistance using the CRISPR/Cas9 system have focused primarily on plant genome modifications (Borrelli et al., 2018; Langner et al., 2018; Yin and Qiu, 2019; Zaynab et al., 2020). In this review, we provide an overview of studies using CRISPR/Cas9-mediated genome editing of host plants and fungal/oomycete pathogens for improving disease resistance. We also describe CRISPR/Cas9 and CRISPR/Cpf1 systems in plants and fungi/oomycetes and conclude with limitations and future perspectives for plant disease resistance through genome editing of host and pathogen.

## Application of CRISPR/Cas9 and CRISPR/Cpf1 Systems in Plant

Unlike TALENs or ZFNs, CRISPR/Cas genome editing is relatively more efficient and convenient, including no need for protein engineering, efficient target cleavage, and utilization of a relatively small protein (Mahfouz et al., 2014). Furthermore, highly feasible multi-target site cleavage and DNA-free delivery of the CRISPR/Cas construct became an exceptional development in the history of genome editing, which can be conducted by delivery of preassembled Cas protein and guide RNA into target organism’s cell (Woo et al., 2015; Zetsche et al., 2017). These beneficial properties provide a more precise and efficient tool for the generation of transgenic non-GMO regulated plants. The most widely characterized and utilized Cas genes are *csn1*, Cas9 (Deltcheva et al., 2011), and *cpf1*, Cas12a (Zetsche et al., 2015). The obvious difference between Cas9 and Cpf1 systems are the RNA complex that binds to each nuclease. The CRISPR/Cas9 system generally uses sgRNA (single guide RNA), which is an artificial chimera carries crRNA (CRISPR RNA), and tracrRNA (trans-activating CRISPR RNA) required for the maturation of crRNA (Deltcheva et al., 2011). The CRISPR/Cpf1 system uses only a crRNA, containing a protospacer and RNA sequences that bind to Cpf1, often called a direct repeat. The minimum length required for RNA to successfully bind to and cleave the target is also different between Cas9 and Cpf1. The minimum guide RNA length for the cleavage of the target using Cas9 is considered 96 bp, including protospacer, but for Cpf1, only 43 bp are required (Xie et al., 2015; Wang et al., 2017). Not only the sgRNA size, but the cleavage mechanisms are also different between Cas9 and Cpf1, which include a variation of the protospacer adjacent motif (PAM) sequence; the Cas9 recognizes 5′-NGG-3′, whereas Cpf1 recognizes 5′-TTN-3′.

**FIGURE 1:**
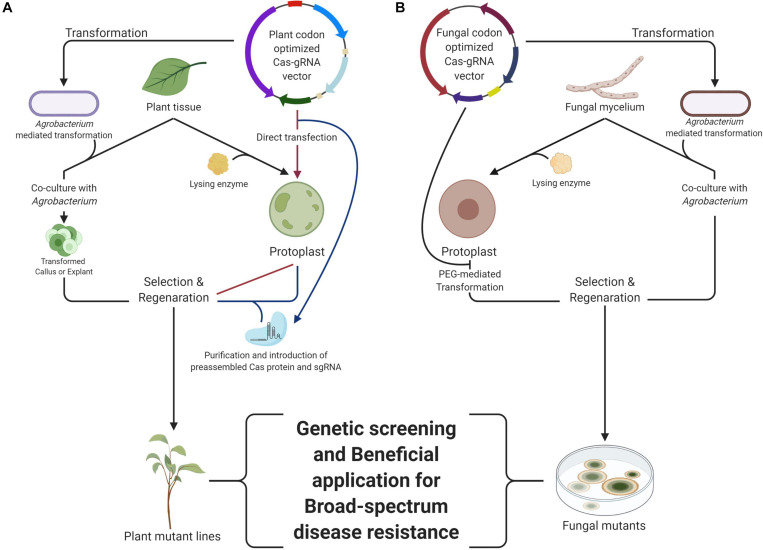
The workflow of CRISPR/Cas system in the plant **(A)** and fungi **(B)**. The *Agrobacterium*-mediated transformation is the common method for genomic modification in plants, including the CRISPR/Cas system delivery. The CRISPR/Cas system is being inserted into the plant genome. The ribonucleoprotein (RNP) delivery into plant protoplast generates no trace except targeted modification in desired loci in the genome. They can be delivered by direct transfection or introduction of a preassembled CRISPR/Cas system. The Polyethylene glycol, PEG-mediated transformation is the common method for genomic modification in fungi, which resembles direct transfection of binary vector to plant protoplast. The *Agrobacterium*-mediated transformation is also optional in the fungal genome modification. With these transformation methods, generation of broad-spectrum resistance can be performed by using each or both mutants. This figure was created with BioRender.com.

Schenke and Cai (2020) summarized that there are several strategies for researching plant disease resistance via the CRISPR/Cas system: (i) knock-out of susceptibility factor-encoding genes (e.g., *MLO*; a mildew resistance locus O) (Nekrasov et al., 2017), (ii) deletion, modification, or introduction of cis-elements in promoters (Oliva et al., 2019), (iii) introducing specific mutations in coding regions via HDR, (iv) alteration of amino acids in plant surface receptor proteins for evasion of secreted pathogen effectors (e.g., *AtBAK1*) (Li et al., 2016), (v) knock-out of negative regulators of plant defense responses (e.g., *TcNPR3*) (Fister et al., 2018), or (vi) modification of central regulators of defense response (e.g., *BnWRKY70*) (Sun et al., 2018). These strategies are applied via three different methods: (i) transformation of Cas and sgRNA constructs into the target plant genome often via *Agrobacterium*-mediated transformation (AMT) (Fister et al., 2018), or (ii) delivering a ribonucleoprotein (RNP) complex consisting of the Cas protein and sgRNA into plant cells for DNA-free plant genome editing via polyethylene glycol (PEG)-mediated protoplast transformation (Figure 1) or (iii) biolistic delivery of RNP complex into plant embryo cells (Svitashev et al., 2016). For the selection of transformants containing the Cas gene and guide RNA expression cassette, a herbicide selective marker, bialaphos resistance gene (*bar*) (Bao et al., 2019), kanamycin resistance gene (*kanR*) (Danilo et al., 2019), or hygromycin resistance gene (*hph*) (Lee et al., 2019) has been used. The various constitutive promoters, such as *Cauliflower mosaic virus* 35S (CaMV 35S), *Arabidopsis thaliana* ubiquitin 1 (*AtUBQ1*), and promoter of meiocyte specific gene *Zea mays* dmc1 (*Zmdmc1*) have been used for the expression of Cas protein. For transcription of guide RNA, *A. thaliana* U6 promoter, *Oryza sativa* U3 promoter, and *Z. mays* U3 promoter have been utilized (Xie and Yang, 2013; Mao et al., 2016; Feng et al., 2018). In plant systems, human or model plant codon-optimized Cas proteins have been used, but it may have affected mutation efficiency in different crops. In addition, the expression of sgRNA is important for successful genome editing. The crop optimized promoters for sgRNA expression have been tested and showed higher efficiency than promoters of model plants in cotton (Long et al., 2018) and grape (Ren et al., 2021), respectively.

## Application of CRISPR/Cas9 and CRISPR/Cpf1 Systems in Fungi and Oomycetes

In fungi and oomycetes, homologous recombination of donor DNA or split marker (for fungi) was previously considered as one of the classical methods for generating mutants, but this method can have the disadvantage of obtaining a small number of mutants (Jeong et al., 2007; Mcleod et al., 2008). Therefore, introducing the donor DNA or split marker together with the CRISPR system was attempted and showed high efficiency (Matsu-ura et al., 2015; Sang et al., 2018; Wang et al., 2019).

For genetic engineering of fungi and oomycetes using the CRISPR system, fungal or human codon optimized Cas9 and Cpf1 sequences have been used. The introduced cassette contains the CRISPR (Cas9 or Cpf1) gene appended with a SV40 or synthetic nuclear localization signal and expression is controlled by a constitutive fungal/oomycete promoter (e.g., *ptef1*, *pgpd*, *pENO1*, *ptrpC*, and *pHam34*) and terminator. SgRNA or crRNA is a guide sequence with a CRISPR target controlled by the promoters of heterologous U6 Pol III, SNR52 promoter, 5s rRNA and tRNA for fungi, and RPL41 promoter for oomycetes, especially *Phytophthora* species (Vyas et al., 2015; Fang and Tyler, 2016; Schuster and Kahmann, 2019). These expression cassettes were delivered by various transformation methods in fungi and oomycetes. For filamentous fungi, there are four commonly used methods: (i) exogenous DNA introduction using PEG solution into protoplasts generated by cell wall lysing enzymes (protoplast mediated transformation, PMT) (Nødvig et al., 2015), (ii) *Agrobacterium tumefaciens* co-cultivation for transferring vector DNA (AMT) (Zou et al., 2020) (Figure 1), (iii) reversible membrane permeabilization induced by local application of electric pulses (Electroporation, EP), and (iv) tungsten particles coated with DNA, accelerated to a high velocity, and introduced into cells (Biolistic transformation, BT) (Wang, 2018). In oomycetes, protoplast- or AMT methods have been mainly used for delivering the CRISPR/Cas9-guide RNA constructs into the cells (Fang and Tyler, 2016; Gumtow et al., 2018).

To efficiently screen the mutants generated by the CRISPR/Cas9 system, a selective marker can be used with the Cas protein and guide RNA sequence cassette. Two types of selective markers are primarily used in fungi: (i) drug resistance markers such as hygromycin-B-phosphotransferase (*hph*, hygromycin resistance), phleomycin resistance protein (*Ble*, phleomycin resistance) (Zhang et al., 2016), or nourseothricin N-acetyl transferase (*NAT*, nourseothricin resistance) (Min et al., 2018), and (ii) auxotrophic markers such as uridine and arginine, *pyrG* (van Leeuwe et al., 2019) and *argB* (Zheng et al., 2017), respectively, have been commonly used across various fungal species. In oomycetes, *hph* is an available selective marker for *P. infestans*, but this marker has low efficiency for other *Phytophthora* species. Recently, oxathiapiprolin resistance gene (*PcMuORP1*) from *P. capsici* has been tested as a novel selective marker and showed high efficiency for *Phytophthora* species transformation (Wang et al., 2019).

## Plant Genome Editing to Improve Disease Resistance Against Fungal Pathogens

Fungal pathogens have an enormous impact on agriculture and represent the dominant causal agents of plant diseases. They cause numerous diseases such as mildews, smuts, blights, rusts, and rots. Additionally, some fungal pathogens also produce mycotoxins, which can cause severe human and animal health problems (Borrelli et al., 2018; Yin and Qiu, 2019; Zaynab et al., 2020). Diverse fungal lifestyles and high genetic flexibility allow fungi to adapt to new hosts, break resistance (R) gene-mediated resistance, and evolve resistance to fungicides, making disease control challenging (Yin and Qiu, 2019).

The CRISPR/Cas9 system has facilitated targeted mutagenesis efficiently and precisely in plants to enhance resistance to fungal diseases. Much attention has been paid to susceptibility genes and negative regulators involved in the defense mechanism for plant disease resistance. The susceptibility gene, a mildew resistance locus O (*MLO*), is the most widely studied gene for resistance to fungal diseases (Jørgensen, 1992). The *MLO* gene encodes an integral membrane protein with seven transmembrane domains and is conserved throughout monocots and dicots (Acevedo-Garcia et al., 2014). Nekrasov et al. (2017) described that there are 16 *MLO* genes in tomato plants and *SlMlo1* is a major gene responsible for powdery mildew disease susceptibility. The CRISPR/Cas9 technology has been employed to knock-out *SlMlo1* in tomato plants. The knock-out mutants conferred resistance to the powdery mildew fungus *Oidium neolycopersici* without generating any other unwanted phenotypic effects. Second generation progeny (F1) were cultivated by selfing the first-generation (F0) resistant mutants, which resulted in the CRISPR/Cas9 transfer DNA being removed via segregation. The F1 progeny also exhibited resistance to *O. neolycopersici*. Likewise, the susceptibility gene Powdery Mildew Resistance 4 (*PMR4*), which functions as a callose synthase, has also been mutated by the CRISPR/Cas9 system, which resulted in resistance to *O. neolycopersici* in tomato (Santillán Martínez et al., 2020). Additionally, in wheat plants mutated by CRISPR/Cas9, one (*TaMLO-A1*) of the three *MLO* homoalleles showed improved resistance to *Blumeria graminis* f. sp. *tritici* infection (Wang et al., 2014). In grapevine, the molecular feasibility of deleting *VvMLO7* has been demonstrated through CRISPR/Cas9 RNP delivery to protoplasts, but no plants have been regenerated (Malnoy et al., 2016). Parallel experiments with RNAi plants showed that the loss of *VvMLO7* reduced susceptibility to *Erysiphe necator* in grapevine (Pessina et al., 2016).

Rice resistance to the rice blast disease caused by *Magnaporthe oryzae* has been improved by targeting negative regulators associated with the defense mechanism using the CRISPR/Cas9 system. This study showed RNAi expression knock-down of the ethylene responsive factor in rice, known as APETELA2/ethylene response factor (AP2/ERF) type transcription factor (*OsERF922*), which resulted in reduced accumulation of abscisic acid and thus increased disease resistance against *M. oryzae* (Liu et al., 2012). Similarly, this gene was targeted by CRISPR/Cas9 in the *japonica* rice variety and T_2_ generation homozygous mutant lines displayed increased resistance to rice blast disease with no difference of agronomic traits with the wild-type rice (Wang et al., 2016). Also, introduced mutations into the coding regions of the thermosensitive male sterile gene *TMS5*, proline-rich protein *Pi21* gene, and bacterial blight resistance a recessive gene *Xa13* in rice via CRISPR/Cas9 improved resistance against rice blast and bacterial blight. This is because the knock-out of recessive genes, *Pi21* and *Xa13*, positively mediated resistance to rice blast and bacterial blight, respectively (Chu et al., 2006; Fukuoka et al., 2009; Li et al., 2019). In grape, the CRISPR/Cas9-mediated targeted mutagenesis of the transcription factor *VvWRKY52* generated biallelic mutation mutant lines and knock-out of *VvWRKY52* enhanced resistance to gray mold disease caused by *Botrytis cinerea* (Wang et al., 2018). In soybean, the novel chromosome rearrangement technique using CRISPR/Cas9 was reported (Nagy et al., 2021). This study engineered two NLR gene clusters called Rpp1-like and Rps1, which related with the resistance for *Phakopsora pachyrhizi* and *P. sojae*, respectively, and showed a possible advanced approach for managing plant disease through artificially creating chimeric paralogs. Together, these studies demonstrated the improved plant resistance against fungal pathogens through mutagenesis of single to multiple CRISPR/Cas9 targets (Table 1).

**TABLE 1:** Applications of CRISPR/Cas9 and CRISPR/Cpf1 systems for plant resistance to fungal and oomycete pathogens.

Fungal or oomycete pathogen	Plant species	Target gene	Encoding protein/function of gene	Disease resistance/phenotype	Method	References
**Plant genome editing**						
*Blumeria graminis* f. sp. *tritici* (Powdery mildew)	*Triticum aestivum* (Wheat)	*TaMLO-A1, B1, D1*	Transmembrane protein/Negative regulator of defense response	Increased resistance to *B. graminis*	Triple-knock-out using particle bombardment of Cas9 and selective marker plasmid	Wang et al., 2014
*Botrytis cinerea* (Gray mold)	*Vitis vinifera* (Grape)	*VvWRKY52*	Transcription factor/Positive regulator of defense response	Increased resistance to *B. cinerea*	*Agrobacterium*-mediated transformation with Cas9/gRNA expression binary vectors	Wang et al., 2018
*Erysiphe necator* (Powdery mildew)	*Vitis vinifera* (Grape)	*VvMLO7*	Transmembrane protein/Negative regulator of defense response	No mutated plants regenerated	Protoplast transformation with Cas9/gRNA expression binary vectors	Malnoy et al., 2016
*Magnaporthe oryzae* (Rice blast)	*Oryza sativa* (Japonica rice)	*OsERF922*	Ethylene responsive factor/Negative regulator of defense response	Enhanced resistance to *M. oryzae*	*Agrobacterium*-mediated transformation with Cas9/gRNA expression binary vectors	Wang et al., 2016
*Magnaporthe oryzae* (Rice blast)	*Oryza sativa* (Japonica rice)	*Pi21*	Negative regulator of defense response	Enhanced resistance to *M. oryzae*	*Agrobacterium*-mediated transformation with Cas9/gRNA expression binary vectors	Li et al., 2019
*Oidium neolycopersici* (Powdery mildew)	*Solanum lycopersicum* (Tomato)	*SlMLO1*	Transmembrane protein/Negative regulator of defense response	Enhanced resistance to *O. neolycopersici*	*Agrobacterium*-mediated transformation with Cas9/gRNA expression binary vectors	Nekrasov et al., 2017
*Oidium neolycopersici* (Powdery mildew)	*Solanum lycopersicum* (Tomato)	*SlPMR4*	Callose synthase gene/Negative regulator of defense response	Enhanced resistance to *O. neolycopersici*	*Agrobacterium*-mediated transformation with Cas9/gRNA expression binary vectors	Santillán Martínez et al., 2020
Pathogenicity not tested	*Ocimum basilicum* (Sweet basil)	*ObDMR1*	Homoserine kinase/Phosphorylation of Homoserine	No infection assay	*Agrobacterium*-mediated transformation with Cas9/gRNA expression binary vectors	Navet and Tian, 2020
*Phytophthora parasitica* (black shank)	*Arabidopsis thaliana* (Arabidopsis)	*AtERF019*	Ethylene responsive factor/Negative regulator of defense response	Enhanced resistance to *P. parasitica*	*Agrobacterium*-mediated transformation with Cas9/gRNA expression binary vectors	Lu et al., 2020
*Phytophthora tropicalis* (black pod)	*Theobroma cacao* (Cacao)	*TcNPR3*	NPR protein family/Negative regulator of defense response	Enhanced resistance to *P. tropicalis*	*Agrobacterium*-mediated transformation with Cas9/gRNA expression binary vectors	Fister et al., 2018
*Plasmopara viticola* (Downy mildew)	*Vitis vinifera* (Grapevine)	*VvPR4b*	Chitinase/Inhibition of fungal mycelium growth	Decreased resistance	*Agrobacterium*-mediated transformation with Cas9/gRNA expression binary vectors	Li et al., 2020
*Sclerotinia sclerotiorum* (stem rot)	*Brassica napus L.* (Rapeseed)	*BnWRKY70*	Central regulator of salicylic acid and jasmonic acid signal pathways.	Enhanced resistance to *S. sclerotiorum*	*Agrobacterium*-mediated transformation with Cas9/gRNA expression binary vectors	Sun et al., 2018
**Fungi/oomycete genome editing**						
*Claviceps purpurea*	*Secale cereale* (Rye)	*CpTrpE, Cppyr4*	α-subunit of anthranilate synthase/Key enzyme of tryptophan biosynthesis orotidine 5’-phosphate decarboxylase/Involved in pyrimidine biosynthesis	Decreased virulence	CRISPR/Cas9 and Homologous recombination (HR) knock-out	Králová et al., 2021
*Leptosphaeria maculans*	*Brassica napus* (rapeseed)	*AvrLm7*	Effector protein/Recognized by host R gene	Enhanced virulence	CRISPR/Cas9	Zou et al., 2020
*Peronophythora litchii*	*Litchi chinensis* (Litchi)	*PlPAE5*	Pectin acetylesterase/Consume host pectin as carbon source	Less invasion	CRISPR/Cas9	Kong et al., 2019
*Phytophthora sojae*	*Glycine max* (Soybean)	*PsSu(z)12*	Core subunit of H3K27me3 methyltransferase/Participate in gene silencing	Induces immune recognition by soybeans	CRISPR/Cas9	Wang et al., 2020
*Phytophthora sojae*	*Glycine max* (Soybean)	*PsAvr3c*	RxLR effector/Promotes host susceptibility	Decreased aggressiveness	CRISPR/Cas9	Huang et al., 2017
*Phytophthora sojae*	*Glycine max* (Soybean)	*Avr4/6*	RxLR effector/Promotes host susceptibility	Evasion of R gene mediated resistance in Rps soybean line	CRISPR/Cas9	Fang and Tyler, 2016
*Phytophthora palmivora*	*Carica papaya* (Papaya)	*PpalEPICs*	Cysteine protease inhibitor/Inhibition of Cysteine protease in host	Induced disease resistance in papaya	CRISPR/Cas9 and *Agrobacterium*-mediated transformation	Gumtow et al., 2018

## Plant Genome Editing to Improve Disease Resistance Against Oomycete Pathogens

The diseases caused by oomycetes include blights, mildews, damping-off, and root rots. They resemble filamentous fungi but are taxonomically different and are more closely related to diatoms and brown algae. The impact of oomycetes on humankind is well documented as both a persistent threat to subsistence and commercial farming as destructive pathogens of native plants and represent a recurring threat to global food security (Kamoun et al., 2015; Derevnina et al., 2016). Among the top 10 oomycete pathogens, *Phytophthora* represents seven species, followed by *Pythium*, *Hyaloperonospora* and *Albugo* species (Kamoun et al., 2015).

CRISPR/Cas tools have been successfully established to reduce oomycete diseases. The knock-out of the PAMP-triggered immunity repressor *AtERF019* (ethylene-responsive factor 19 gene) in *A. thaliana* via CRISPR/Cas9 increased resistance to *Phytophthora parasitica*, a model organism of *Phytophthora* species (Lu et al., 2020). The *ObDMR1* knock-out mutants of sweet basil (*Ocimum basilicum*) were generated, but pathogenicity tests in those mutants were not conducted (Navet and Tian, 2020). In the cacao tree (*Theobroma cacao*), genome editing was conducted using *Agrobacterium*-mediated transient transformation to introduce CRISPR/Cas9 components that targeted non-expressor pathogenesis-related 3 (*TcNPR3*), a suppressor of the defense response, into cacao leaves and cotyledon cells. The *TcNPR3* deleted leaves exhibited up-regulated expression of defense genes, such as pathogenesis related (PR) genes, and enhanced resistance to the black pod disease caused by *Phytophthora tropicalis* (Fister et al., 2018). Lastly, the coding region of pathogenesis-related 4 protein genes of *Vitis vinifera* (*VvPR4b*) in grape was knocked-out, and these mutant lines displayed increased susceptibility to downy mildew, confirming that the gene plays an active role in the defense of grapevine against downy mildew (Li et al., 2020) (Table 1).

## Fungal and Oomycete Genome Editing Using CRISPR/Cas9 for Disease Resistance Study

Diverse strategies are being applied to prevent or control plant diseases. With the development of CRISPR/Cas9, editing the genomes of fungal and oomycete species for various purposes also can provide new strategies for plant disease management. Mutants of plant pathogens with variable pathogenicity from wild type can be generated using the CRISPR/Cas9 system. Zou et al. (2020) described a point mutation in the avirulence gene of *Leptosphaeria maculans* 7 (*AvrLm7*) that resulted in a shift from an avirulent strain (*UMAvr7*) to a virulent strain (*umavr7*) on the *Brassica napus* Rlm7 genotype. This pathogen *L. maculans* causes a severe disease called blackleg in canola (*B. napus*). Resistance-susceptibility evaluation based on this mutant strain revealed that six of the 123 *B. napus* genotypes showed resistance to this strain. This mutant generated by the CRISPR/Cas9 system helped to discover new resistance genotypes and potentially novel resistance genes. For the ergot fungus, a widespread biotrophic plant pathogen *Claviceps purpurea*, the CRISPR/Cas9 method was used for directed mutagenesis targeting pyrimidine biosynthesis and tryptophan biosynthesis genes (*pyr4* and *TrpE)*. The *TrpE* mutants showed no infection to rye plants because of a reduction of the plant hormone auxin, which is normally synthesized by *C*. *purpurea* in *Trp*-dependent and *Trp*-independent biosynthetic pathways, ultimately affecting the ability of the fungus to colonize rye plants (Králová et al., 2021).

It is well known that oomycete pathogens secrete numerous effector proteins into host tissues to suppress host defense responses and promote effective colonization. Due to inherent characteristics, oomycetes being diploid, polyploid, or heterothallic, genetic identification of their virulence genes had been very challenging until the recent emergence of CRISPR/Cas9-mediated gene-editing technology (Fang and Tyler, 2016; Gumtow et al., 2018). Fang and Tyler (2016) used the technology to validate the contribution of the *P. sojae* RXLR effector *Avr4/6* in the recognition of the soybean resistance gene loci, *Rps4* and *Rps6*. By knocking out *PsAvr3c* in *P*. *sojae* using the CRISPR/Cas9 system, it was observed that the mutants could infect soybeans with the *Rps3c* resistant gene and that *PsAvr3c* is required for full virulence of *P*. *sojae* (Huang et al., 2017). It was also found that *P. sojae* evades the soybean resistance gene *Rps1b* through silencing of *Avr1b* by epigenetic modification. After editing *PsSu(z)12*, which encodes a core subunit of the H3K27me3 methyltransferase complex, with the CRISPR/Cas9 system, the knock-out mutants lost the ability to avoid immune recognition by soybeans carrying *Rps1b* (Wang et al., 2020). Knock-out of pectin acetylesterases (*PlPAE4* and *PlPAE5*) from *Peronophythora litchi*, the causal agent of the downy blossom blight, using the CRISPR/Cas9 system is another example. The *PLPAE5* knock-out mutants were less capable of host invasion than the wild-type strain, which suggested that *PlPAE5* is involved in oomycete pathogenicity (Kong et al., 2019). In the papaya pathogen *Phytophthora palmivora*, homozygous *PpalEPIC8* mutants were generated using the CRISPR/Cas9 system, and it was found that the mutants exhibited reduced pathogenicity on papaya fruits. This is because knock-out of the cysteine protease inhibitor *PpalEPC8* reduced the inhibition of papain in papaya, a cysteine protease that confers defense against plant pathogens (Gumtow et al., 2018) (Table 1).

## Limitations of CRISPR/Cas9 and Future Perspectives

The increasing availability of crop and fungal/oomycete genome sequences and user-friendly CRISPR/Cas9 tools with open resources (e.g., protocols and plasmids) have accelerated the development of disease resistance in diverse crops. However, there are few limitations that will need to be addressed. For example, the relatively long-life cycle of crop plants, in comparison to model plants, makes accuracy and frequency of mutant generation a significant issue. The recent application of the CRISPR/Cas9 system through agroinfiltration to cacao was tested and succeeded in showing a disease resistance phenotype (Fister et al., 2018). Therefore, increased research into the fast and convenient ways to confirm the resistance phenotype by transient expression of CRISPR/Cas9 components are needed. For fungi/oomycetes, more fundamental difficulties limit genome editing, such as lack of selectable markers. Recently, the selectable marker (oxathiapiprolin-resistance gene) for *Phytophthora* sp. was developed and has shown a considerably lower minimal inhibitory concentration than geneticin (G418) or hygromycin B (Wang et al., 2019), but other oomycetes have not been studied yet.

The side effects of editing or mutating plant susceptibility genes should be considered before applying CRISPR/Cas9 genome editing techniques. It is known that the susceptibility gene(s) are linked with plant growth and fitness and can cause pleiotropic effects (Tyagi et al., 2021). This is also a most frequently observed phenomenon after artificially boosting plant immunity through knock-out of a negative regulator (Ding et al., 2018). Although several studies have indicated that deletion or mutation of these genes do not have any negative impact on plant growth or plant health (Pyott et al., 2016), further studies and experimentation are needed. Additionally, introduction of off-target mutations by the CRISPR/Cas9 system is an important issue to consider. An off-target mutation is an undesirable result that causes non-specific changes in the genome sequences that ultimately influences protein structure and/or function. Currently, scientists are working on reducing off-target issues by using computational tools (Guide-seq, Diagenome-seq, DISCOVER, etc.) and by re-editing the CRISPR components such as Cas proteins and gRNA (Ahmad et al., 2020).

Genome editing by CRISPR/Cas9 could produce desired disease-resistance within a short period compared to traditional or molecular breeding methods. However, single gene-editing based disease resistance can eventually be overcome by pathogens due to rapid evolution and genetic diversity of fungal and oomycete populations. For long-term disease management, targeting multiple genes and/or the combined editing of both plant and fungal/oomycete genomes using these rapid-developing CRISPR/Cas9 technologies could be powerful approaches. One concept that can be demonstrated by combined genome editing is the interaction between a pathogen effector and host plant resistance gene. For example, editing of the *P. sojae* effector *Avr* genes (Fang and Tyler, 2016) and the soybean *Rps* genes (Nagy et al., 2021) can be combined and applied for controlling Phytophthora root and stem rot in soybean. Also, one example that can be applied to plant disease control is eradicating malaria vector mosquitoes by the targeted disruption of the doublesex gene using the CRISPR/Cas9 gene drive system (Kyrou et al., 2018). This gene drive system could be a model to control sexually inheriting plant pathogens. Application of the CRISPR/Cas9 system to beneficial fungal species, such as *Trichoderma* sp., to enhance plant immunity and function as a biocontrol agent against fungal and oomycete pathogens is also a promising approach. CRISPR/Cas9-based plant and pathogen genome editing will likely be widely adopted to enhance plant disease-resistance, and the disease-resistant transgene-free crops will be necessary for global demand for food in the future.

## Author Contributions

HS conceptualized, designed, edited, managed, and acquired funding to conduct the research. NCP and S-WP contributed equally to the writing of the manuscript. HL, SC, JM, JSM, and MC wrote and edited part of the manuscript. All authors reviewed and edited and have read and agreed to submit the final version of the manuscript.

## Conflict of Interest

The authors declare that the research was conducted in the absence of any commercial or financial relationships that could be construed as a potential conflict of interest.

## Publisher’s Note

All claims expressed in this article are solely those of the authors and do not necessarily represent those of their affiliated organizations, or those of the publisher, the editors and the reviewers. Any product that may be evaluated in this article, or claim that may be made by its manufacturer, is not guaranteed or endorsed by the publisher.
